# Impact of stereotactic body radiotherapy vs palliative radiotherapy on oncologic outcomes of patients with metastatic kidney cancer concomitantly treated with immune checkpoint inhibitors: a preliminary, multicentre experience

**DOI:** 10.1007/s12094-022-02844-5

**Published:** 2022-06-23

**Authors:** Giulio Francolini, Riccardo Campi, Vanessa Di Cataldo, Beatrice Detti, Mauro Loi, Luca Triggiani, Salvatore La Mattina, Paolo Borghetti, Stefano Maria Magrini, Luca Nicosia, Filippo Alongi, Paolo Ghirardelli, Vittorio Vavassori, Andrea Gaetano Allegra, Michele Aquilano, Erika Scoccimarro, Anna Peruzzi, Pierpaolo Pastina, Luca Visani, Isacco Desideri, Sergio Serni, Icro Meattini, Lorenzo Livi

**Affiliations:** 1 Radiotherapy Unit, Azienda Ospedaliera Universitaria Careggi, Florence, Italy; 2 Unit of Urological Robotic Surgery and Renal Transplantation, University of Florence, Careggi Hospital, Florence, Italy; 3 Radiotherapy Department, Istituto Fiorentino di Cura e Assistenza, Florence, Italy; 4 Department of Radiation Oncology, University and Spedali Civili Hospital, Brescia, Italy; 5 Radiation Oncology, IRCCS Sacro Cuore Don Calabria Hospital, Negrar, Verona, Italy; 6 Department of Radiotherapy, Cliniche Humanitas Gavazzeni, Bergamo, Italy; 7 Department of Biomedical, Experimental and Clinical Sciences “Mario Serio”, University of Florence, Florence, Italy; 8 Section of Radiation Oncology, Medical School, University of Siena, Siena, Italy; 9 Department of Experimental and Clinical Medicine, University of Florence, Florence, Italy

**Keywords:** Metastatic renal cell carcinoma, SBRT, Stereotactic ablative radiotherapy

## Abstract

**Purpose:**

To explore the benefit yielded by radiotherapy (RT), we report a series of metastatic renal cell carcinoma (RCC) patients treated with concomitant RT plus Nivolumab.

**Methods/patients:**

Patients undergoing Nivolumab treatment plus concomitant RT (ablative or palliative) were included. RT was defined Ablative if >5 Gy/fraction were delivered.

**Results:**

Ablative RT intent was the only independent predictor of both progression free and overall survival (HR 3.51, 95% CI 1.6–7.5, *p* = 0.0012 and HR 2.8, 95% CI 0.99–8.07, *p* = 0.05, respectively).

**Conclusion:**

Ablative RT may improve oncologic outcomes in selected patients with metastatic RCC treated with Nivolumab as compared to palliative RT.

## Introduction

Immune checkpoint inhibition (ICI) is one of the cornerstones of the contemporary treatment of metastatic renal cell carcinoma (RCC), both in first [1–4] and second line settings [5, 6]. Notably, the latest European Association of Urology (EAU) Guidelines recommend to offer stereotactic body radiotherapy (SBRT) to patients with metastatic disease and favorable disease factors to control local symptoms [6]. Interestingly, pre-clinical evidence highlighted a biological rationale for the potential added benefit of SBRT on top of ICI, while clinical evidence of such a synergistic effect is still controversial [7]. Herein, we report a retrospective multicentric series of metastatic RCC patients treated with concomitant radiotherapy (RT) plus ICI (Nivolumab as I-, II- or III-line therapy), aiming to compare the benefit yielded by SBRT vs palliative RT in this setting.

## Material and Methods

After Ethical Committee approval, data from patients treated between June 2016 and November 2020 at 3 referral centers were retrospectively collected. All included patients gave written consent. Patients with either synchronous or metachronous metastatic RCC undergoing ICI with Nivolumab as I- or II-/III-line treatment plus *concomitant* RT were included. RT was considered concomitant if it was administered within ≤ 4 months before the start or after the end of Nivolumab treatment.

**Fig. 1:**
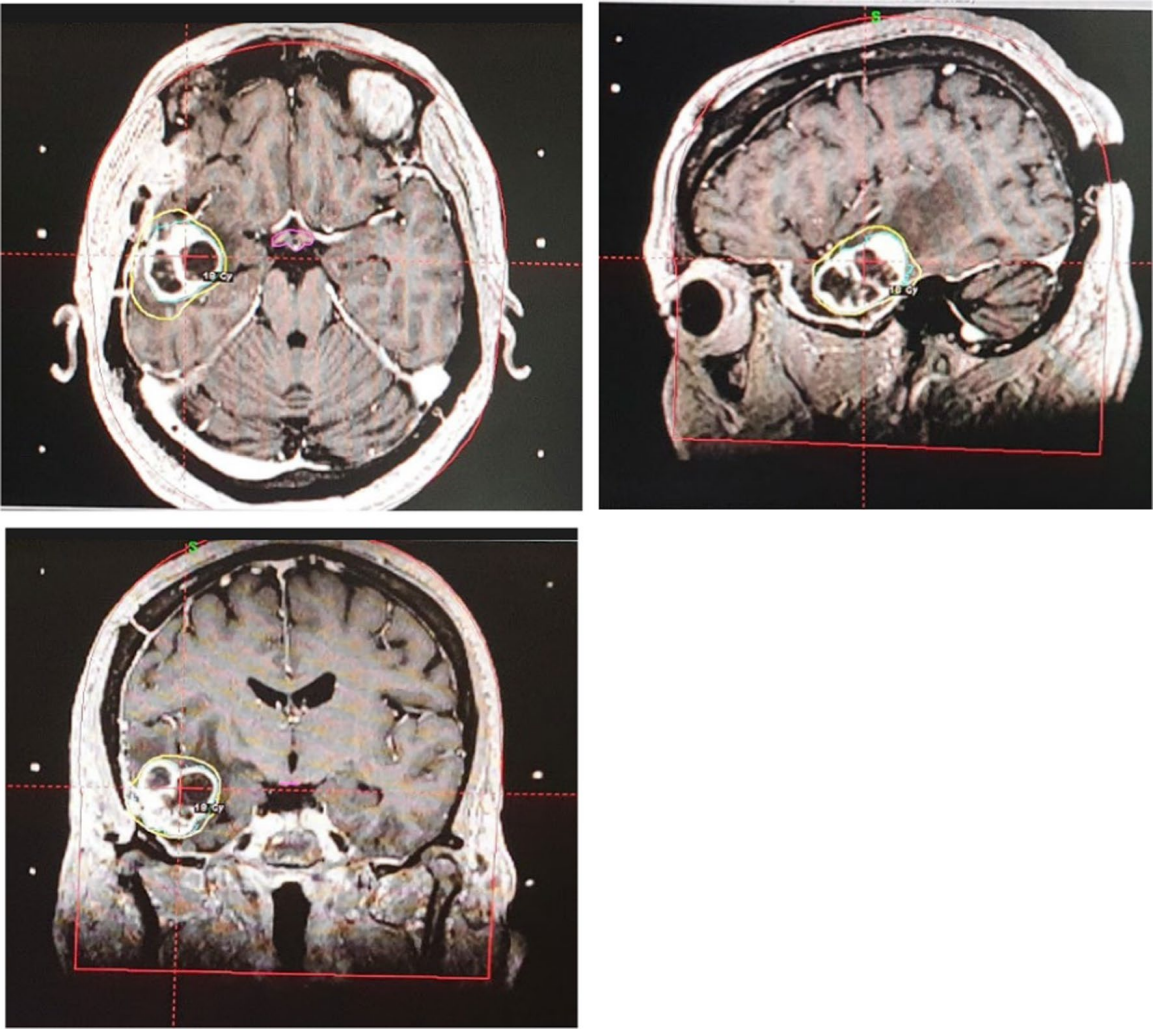
Example of a metastatic brain lesion treated with ablative intent (18 Gy in 1 fraction)

RT was administered with either an *ablative* (SBRT) or palliative treatment purpose based on each patient’s characteristics (i.e., metastatic site and technical feasibility of an ablative approach) according to physicians’ discretion. RT regimens were chosen according to adjacent critical organs at risk, and were defined as ablative if ≥ 5 Gy/fraction were delivered [8]. SBRT included regimens providing total doses of 18–54 Gy in 1–8 fractions, while 20–39 Gy in 5–15 fractions were administered for palliative treatments. Doses and fractionation schedules were prescribed according to clinician choice in the ablative group aiming to administer the maximum equivalent dose to the target respecting organ at risk dose constraints, with at least 5 GY per fraction. In patients undergoing palliative treatment, dose and fractionation schedules were delivered aiming to administered a dose at least equivalent to 20 Gy in 5 fraction considering an alpha/beta ratio of 10 (Fig. 1). Volumetric Modulated Arc Therapy and 3d Conformal technique were used for palliative purpose, Ablative treatment were administered also with Cyberknife^R^ robotic system or Gammaknife^R^ (Table 1). Detail for doses and fractionation schedules for 9 brain metastases treated are summarized in Table 2.

**Table 1:** Main dose/fractionation schedules and techniques used for 52 treated lesions

Dose/fractionation	*n* (%)
Ablative treatments	28 (53.8)
44 Gy/8 fractions	1 (1.9)
36 Gy/6 fractions	2 (3.9)
45 Gy/5 fractions	1 (1.9)
40 Gy/5 fractions	1 (1.9)
30 Gy/5 fractions	10 (19.3)
25 Gy/5 fractions	1 (1.9)
54 Gy/3 fractions	1 (1.9)
45 Gy/3 fractions	1 (1.9)
30 Gy/3 fractions	1 (1.9)
18 Gy/3 fractions	1 (1.9)
24 Gy/1 fraction	8 (15.4)
Techniques	
VMAT	20 (38.5)
GK	6 (11.5)
CK	2 (3.8)
Palliative treatments	24 (46.2)
37.5 Gy/15 fractions	1 (1.9)
39 Gy/13 fractions	1 (1.9)
30 Gy/10 fractions	5 (9.7)
20 Gy/5 fractions	17 (32.7)
Techniques	
VMAT	4 (7.7)
3dCRT	20 (38.5)

*VMAT* volumetric modulated arc therapy, *GK* Gammaknife radiosurgery, *CK* Cyberknife^R^ robotic radiotherapy, *3dCRT* three dimensional conformal radiotherapy

**Table 2:** Main dose/fractionation schedules and techniques used for 9 brain treated lesions

Dose/fractionation	*n *(%)
Brain treatments	9 (17.3)
24 Gy/single fraction	5 (9.6)
21 Gy/single fraction	1 (1.9)
20 Gy/single fraction	1 (1.9)
18 Gy/single fraction	1 (1.9)
18 Gy/3 fractions	1 (1.9)
Techniques	
VMAT	2 (3.8)
GK	6 (11.5)
CK	1 (1.9)

VMAT volumetric modulated arc therapy, GK Gammaknife radiosurgery, CK Cyberknife^R^ robotic radiotherapy

The primary outcomes of the study were overall survival (OS) and progression-free survival (PFS). Overall survival (OS) was defined as time between Nivolumab Start and death. Progression-free survival (PFS) was defined as time between Nivolumab start and end. Local control outcomes and adverse events according to Common Terminology Criteria for Adverse Events were collected and reported. Kaplan–Meier analysis was performed to explore the correlation between clinical outcomes, initial staging (metachronous vs synchronous metastases) and RT intent (ablative vs palliative only). Cox proportional hazard model was used for multivariate analysis, including stage at diagnosis (metastatic vs non-metastatic) and RT goal (ablative vs palliative). Chi square test was used to compare local control in patients treated with ablative and palliative intent.

## Results

**Fig. 2:**
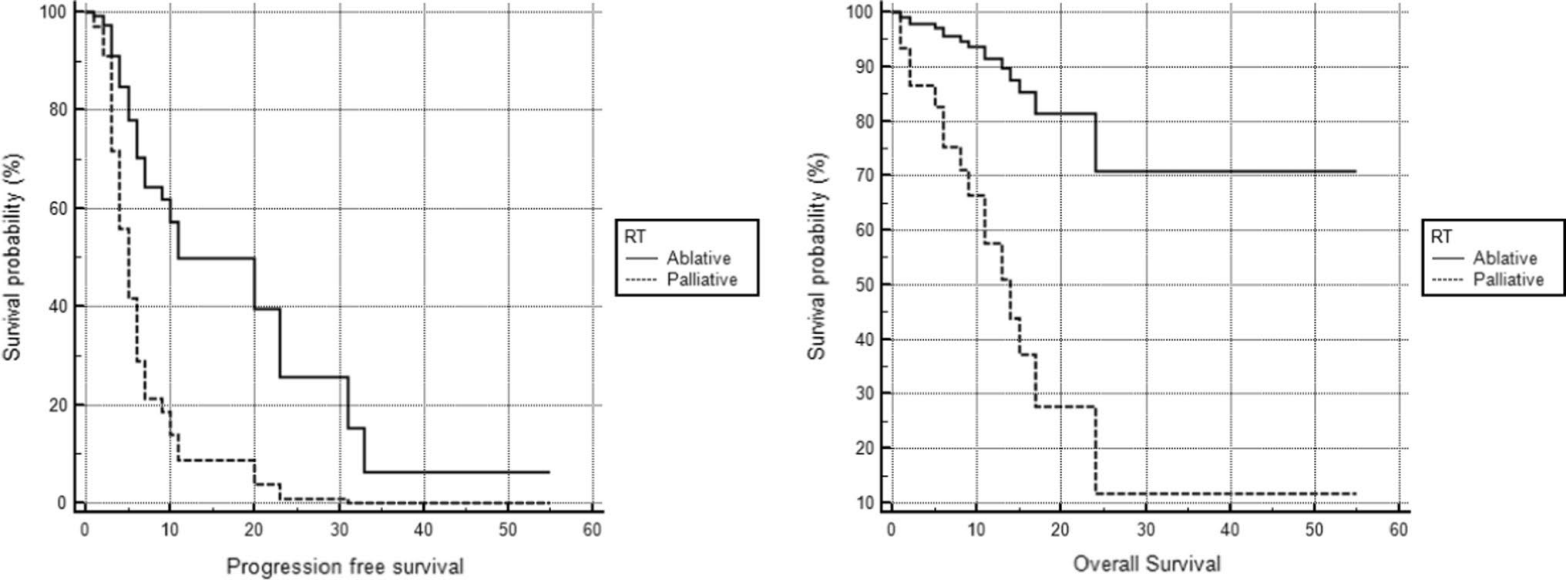
Cox proportional hazard model for progression free and overall survival

Overall, 40 patients with 52 metastatic lesions were included, 19 (47.5%) were treated with Nivolumab plus SBRT, and 21 (52.5%) with Nivolumab plus palliative RT. Baseline patient characteristics are summarized in Table 3. The proportion of patients with IMDC poor/intermediate risk was comparable between the study groups (*p* = 0.43). Among patients with synchronous metastatic RCC (*n* = 15), 14 (93.3%) underwent cytoreductive nephrectomy (CN) before ICI plus RT treatment. After a median follow-up of 11 months (IQR 4.7–17.6), 16 patients died. Overall median PFS and OS were 6 months (95% CI 5–10) and 24 months (95% CI 13–24), respectively. Local progression occurred in 14 treated lesions (26.9%), 5 and 9 in the ablative and palliative RT group, respectively. Local control was significantly improved for lesions treated with ablative if compared to palliative intent (71.8 vs 37.5%, *p* = 0.0009). Distant progression occurred after treatment of 39 out of 52 lesions (75%), 19 and 20 in the ablative and palliative RT group, respectively. No difference in terms of distant control was detected for lesions treated with ablative if compared to palliative intent (32.1 vs 16.6%, *p* = 0.19). The absence of metastasis at diagnosis and ablative RT intent were significantly associated with improved PFS (9 vs 4 months, *p* = 0.005 and 20 vs 5 months, *p* < 0.0001, respectively). Metachronous metastases and concomitant ablative RT were also predictive of improved OS (not reached vs 11 months for both, *p* = 0.02 and *p* = 0.001, respectively). At multivariable Cox regression analysis, ablative RT intent was the only independent predictor of both PFS and OS (HR 3.51, 95% CI 1.6–7.5, *p* = 0.0012 and HR 2.8, 95% CI 0.99–8.07, *p* = 0.05, respectively) (Fig. 2). The overall toxicity profile of both SBRT and palliative RT was mild and principally related to ICI administration, with > G2 events occurring in 14 patients overall (7 endocrine, 2 skin rash, 1 pneumonitis, 3 hepatic, and 2 pancreatic).

**Table 3:** Principal baseline features of patients included

Characteristics	*n* (%)
RT intent	
Ablative	19 (47.5)
Palliative	21 (52.5)
Initial staging	
Synchronous metastases	15 (37.5)
Metachronous metastases	25 (62.5)
Metastatic site	
Bone	22 (55)
Brain	7 (17.5)
Visceral	7 (17.5)
Nodal	3 (7.5)
Soft tissues	1 (2.5)
IMDC risk group	
Favorable	15 (37.5)
Intermediate/poor	25 (62.5)
Cytoreductive nephrectomy^a^	
Yes	14 (93.3)
No	1 (6.7)
Treatment lines before Nivolumab	
0	5 (12.5)
1	25 (62.5)
≥ 2	10 (25)

^a^Percentages are related to patients with synchronous metastasis at diagnosis

## Discussion

While limited by its retrospective nature, small sample size, relatively short follow-up, selection bias and confounding, our preliminary experience outlines the potential benefit of SBRT vs palliative RT for patients with both synchronous (after CN) and metachronous metastatic RCC treated with ICIs. In addition, it highlights the current unmet clinical need of exploring the indications and outcomes of multimodal treatment (i.e., ICI-based systemic therapy + / − CN + / − SBRT) in well-selected patients with metastatic RCC treated by multidisciplinary tumour boards.

In our study, ablative SBRT + ICI yielded significantly better oncologic outcomes in terms of PFS and OS and a higher degree of local control of metastatic lesions as compared to palliative RT. Of note, despite the promising impact on clinical history of these patients, no evidence of the so-called “abscopal effect” (increase in distant control in patients treated with SBRT) was noticed, raising concerns regarding its suitability as a key endpoint for a prospective clinical trial.

Our findings are consistent with an increasing body of evidence showing the potential added value of ablative RT in patients treated with ICI. In fact, in the RADVAX trial enrolling patients with RCC treated with ICI and concomitant SBRT, the overall response rate was 56% [9]. Similar results were found in the RAPPORT trial, enrolling patients treated with RT (either SBRT or conventional palliative RT if SBRT was not feasible) followed by pembrolizumab [10]; yet, data on the differential outcomes between ablative and palliative RT were not available.

## Conclusion

In conclusion, our experience suggests that SBRT may improve local control and oncologic outcomes in carefully selected patients with metastatic RCC treated with ICIs as compared to palliative RT. In light of our study design, our findings are hypothesis-generating and should prompt the design of larger prospective clinical trials evaluating the added value of ablative RT in this setting.
